# Phenolic Compounds from An Algerian Endemic Species of *Hypochaeris laevigata* var. *hipponensis* and Investigation of Antioxidant Activities

**DOI:** 10.3390/plants9040514

**Published:** 2020-04-16

**Authors:** Nabila Souilah, Zain Ullah, Hamdi Bendif, Kamel Medjroubi, Tahar Hazmoune, Tarek Hamel, Mehmet Öztürk, Gema Nieto, Salah Akkal

**Affiliations:** 1 Laboratory of Optimization of Agricultural Production in Sub-Humid Zones (LOPAZS), Faculty of Science, University of Skikda, Skikda 21000, Algeria; souilah_n_phyto@hotmail.fr (N.S.); hazmoune_tahar@yahoo.fr (T.H.); 2 Laboratory of Valorization of Natural Resources, Bioactive Molecules and Physicochemical and Biological Analyzes Department of Chemistry, Faculty of Exact Sciences, University of Constantine 1, Constantine 25000, Algeria; medjroubi@yahoo.fr (K.M.); salah4dz@yahoo.fr (S.A.); 3 Department of Chemistry, Faculty of Sciences, University of Muğla Sitki Koçman, Muğla 48000, Turkey; zainaup614@yahoo.com (Z.U.); mehmetozturk@mu.edu.tr (M.Ö.); 4 Department of Natural Sciences and Life, Faculty of Science, University of M’sila, M’sila 28000, Algeria; hamdi.bendif@univ-msila.dz; 5 Laboratoire d’ethnobotanique et des substances naturelles, Département des sciences naturelles, Ecole Normale Supérieure (ENS), Kouba, BP 92 Kouba, Algiers 16308, Algeria; 6 Department of Natural Sciences and Life, Faculty of Science, University of Badji Mokhtar, Annaba 23000, Algeria; tarek_hamel@yahoo.fr; 7 Department of Food Technology, Food Science and Nutrition, Faculty of Veterinary Sciences, Regional Campus of International Excellence “Campus Mare Nostrum”, Espinardo, 30071 Murcia, Spain

**Keywords:** *Hypochaeris laevigata* var. *hipponensis*, Asteraceae, phenolic compounds, antioxidants activities, LC-MS/MS

## Abstract

*Hypochaeris laevigata* var. *hipponensis* (Asteraceae) is an endemic plant from Algeria. In the current study, we analyzed for the first time its chemical composition, especially phenolic constituents of dichloromethane (DCM), ethyl acetate (EA), and n-butanol (BuOH) fractionsof the aerial parts of *Hypochaeris laevigata* var. *hipponensis* by liquid chromatography-mass spectrometry (LC-MS/MS). The number of phenolic compounds detected in DCM, EA, and BuOH fractions were found to be 9, 20, and 15, respectively. More specifically, 12 phenolic acids were detected. Among them, quinic acid, chlorogenic acid, and caffeic acid were the most abundant ones. Meanwhile, only seven flavonoids were detected. Among them, rutin, apigetrin, and isoquercitrin were the major ones. We also determined the total phenolic and flavonoid contents, and fraction EA showed the highest values, followed by BuOH, and DCM fractions. Furthermore, the antioxidant action was dictated by five methods and the tested plant fractions demonstrated a noteworthy antioxidant action.

## 1. Introduction

A large number of medicinal and aromatic plants grow spontaneously in the Edough Peninsula, such as plants of family Asteraceae which are rich in phenolic compounds, volatile oils, and other bioactive compounds. It is fundamental to extend the knowledge of the chemical composition of some plants of this family [1]. 

According to Stebbins [2], *Hypochaeris* is a smallgenusof the Asteraceae family, which contains about 50 species. On the other, the genus of *Hypochoaeris* contains 100 species, the majority of which are native to South America. The species of *Hypochaeris laevigata* var. *hipponensis* is a perennial plant with a bitter root, endemic to Algeria, but very common everywhere, and on the coast usually develops on wet rocks [3] and is used as a salad by the local population of Sérraidi. 

Nowadays, no studies have been conducted regarding the phytochemicalcomposition of *Hypochaeris laevigata* var. *hipponensis*, except that of Jamunaet al. [4] who studiedthe composition of the species *H. radicata* and reported the presence of alkaloids, flavonoids, glycosides, cardiac glycosides, phenols, resins, saponins, steroids, tannins, terpenoids, and triterpenoids. *Hypochaeris radicata* is medically important and has anti-inflammatory, anticancer, antioxidant [5], antibacterial [6], antifungal [7] properties, and antidiuretics. It is used for the treatment of jaundice, rheumatism, dyspepsia, constipation, hypoglycemia, and kidney problems in the traditional medicinal practice of Tamil Nadu, India [8]. However, no scientific validation has been made for this species for medicinal purposes.

The aim of the present work was to study the chemical composition of *Hypochaeris laevigata* var. *hipponensis,* which is an endemic species from Algeria that has not been reported before, and to evaluate the phenolic compounds of the plant by the liquid chromatography-mass spectrometry (LC-MS/MS) technique and its antioxidant activities.

## 2. Materials and Methods

### 2.1. Plant Material and Extraction Method 

**Figure 1:**
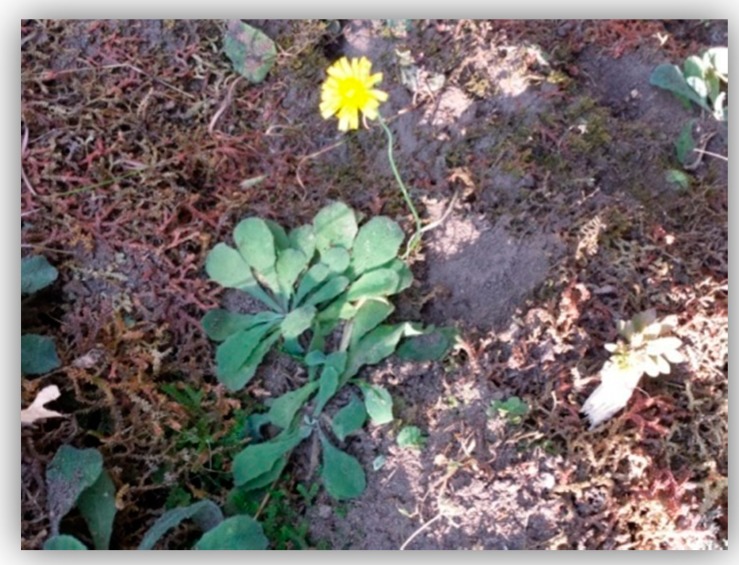
The plant of *Hypochaeris laevigata* var. *hipponensis*.

A sample of the whole plant (*Hypochaeris laevigata* var. *hipponensis*) iscollected in full bloom in Sérraïdi (Annaba), in northeastern Algeria in May 2015 (Figure 1). The plant was identified by Dr. Tarek Hamel, Lecturer at the Department of Plant Biology and Environment, Badji Mokhtar University (Annaba, Algeria). A reference specimen was deposited in the herbarium of the laboratory under the reference code: ChifaDZUMCAPBC000038. 

The aerial part of the plant (800 g) was dried in the shade at room temperature in a ventilated place, cut into small pieces and macerated in a mixture of methanol/water (70/30, *v/v*) at a ratio of 1:10 (*w/v*) for 24 h with a constant stirring speed of 200 rpm, at room temperature. The suspension was then filtered on whatman paper. The extraction is repeated three times till exhaustion, then the solvent was evaporated at 40 °C using Rota Vapor (Büchi R-200, Aachen, Germany) to afford 3.73% of crude extract. 

The crude extract was dissolved in 90% aqueous methanol for fractionation with different solvents such as dichloromethane (DCM), ethyl acetate (EA) and n-butanol (BuOH). Briefly, first fractionation was carried out with 100 mL DCM three times. DCM fraction was collected and evaporated under reduced pressure to give a semi-solid paste. Then, the residual aqueous phase of dichloromethane was further fractionated with EA and BuOH solvents. The resulting fractions were evaporated to dryness. Theyields of DCM, EA, and BuOHfractionswerefound to be1.09%, 0.79%, and 1.63%, respectively. Dried fractions were dissolved in methanol and kept at a temperature of 4 °C for further analysis.

### 2.2. Preparation of Standards

The standard stock solutions were prepared in methanol (50 μg/mL) except hesperidin and isoquercitrin that were dissolved in dimethyl formamide (50 μg/mL). From the stock solutions, a number of working solutions were prepared by appropriate dilution in methanol. All solutions were stored in a refrigerator at 4 °C until analysis.

### 2.3. LC-MS/MS Analysis

The LC-MS analyses of phenolic compounds were performed using a Nexera model Shimadzu UHPLC coupled to a tandem MS instrument. The liquid chromatography was equipped with LC30AD binary pumps, CTO-10ASvp column oven, DGU-20A3R degasser and SIL-30AC autosampler. The chromatographic separation was performed on an RP-C18 Inertsil ODS-4 (100 mm × 2, 1 mm, 2 μm) analytical column. Reversed-phase ultrahigh performance liquid chromatography was optimized to achieve optimum separation for 37 phytochemical compounds and to overcome the suppression effects. The column temperature was fixed at 35 °C. The elution gradient consisted of eluent A (water, 10 mM ammonium formate and 0.1% formic acid) and eluent B (acetonitrile). The following gradient elution program was applied: 5%–20% B (0–10 min), 20% B (10–22 min), 20%–50% B (22–36 min), 95% B (36–40 min), 5% B (40–50 min). The solvent flow rate was maintained at 0.25 mL/min and injection volume was settled as 4 μL.

MS detection was performed using a Shimadzu brand LCMS 8040 model tandem mass spectrometer equipped with an ElectroSpray Ionization (ESI) source operating in negative ion mode. LC-ESI-MS/MS data was collected and shipped by LabSolutions Software (Shimadzu) software. Multiple reaction monitoring (MRM) was used to quantify it. The working conditions of the mass spectrometer were passed as interface temperature, 350 °C; DL temperature, 250 °C; temperature of the thermal block, 400 °C; nebulizationgas flow (nitrogen), 3 L/min; and drying gas stream (nitrogen), 15 L/min. Quantification of the target compounds was performed after optimizing the acquisition parameters (Table 1).

**Figure 2:**
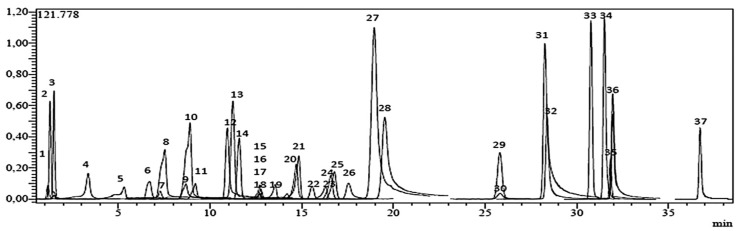
TIC chromatogram of the standards mixture (1 μg/mL) analyzed by the LC-MS/MS. Legend: (**1**) quinic acid, (**2**) malic acid, (**3**) fumaric acid, (**4**) gallic acid, (**5**) protocatechic acid, (**6**) pyrocatechol, (**7**) chlorogenic acid, (**8**) 4-OH-benzoic acid, (**9**) vanillic acid, (**10**) caffeic acid, (**11**) syringic acid, (**12**) vanillin, (**13**) salicylic acid, (**14**) *p*-coumaric acid, (**15**) rutin, (**16**) ferulic acid, (**17**) sinapic acid, (**18**) hesperidin, (**19**) isoquercitrin, (**20**) rosmarinic acid, (**21**) nicotiflorin, (**22**) *α*-coumaric acid, (**23**) rhoifolin, (**24**) quercitrin, (**25**) apigetrin, (**26**) coumarin, (**27**) myricetin, (**28**) fisetin, (**29**) cinnamic acid, (**30**) liquiritigenin, (**31**) quercetin, (**32**) luteolin, (**33**) naringenin, (**34**) apigenin, (**35**) hesperetin, (**36**) kaempferol and (**37**) chrysin.

A complete LC-MS/MS method was optimized and validated for the quantification of 37 phytochemical fingerprint compounds (17 flavonoids, 15 phenolic acids, 3 non-phenolic organic acids, 1 benzopyrene and 1 phenolic aldehyde) on the species studied. The performance characteristics of the method were determined using standard solutions as well as enriched and non-enriched samples. In this context, the developed method has been fully validated in terms of linearity, accuracy (recovery), inter-day and intra-day precision (repeatability), detection and quantification limits (LOD /LOQ) and uncertainty relative standards (U% at 95% confidence level [k = 2]) (Table 2, Figure 2). The dry extracts were prepared at a concentration of 1 mg/mL and filtered with a 0.2 μm syringe filter prior to LC-MS/MS analysis. Each sample was analyzed three times.

### 2.4. Quantification of Total Phenols

The total phenolic content was evaluated according to the method described by Li et al. [9]. Thereby 1.5 mL of the Folin–Ciocalteu reagent previously diluted ten times with distilled water was added to 300 μL of the extract. After 4 min, 1.2 mL of 7.5% sodium carbonate (Na_2_CO_3_) was poured onto the solution. The samples were placed in the dark. After 2 h, the results were read on a spectrophotometer at 750 nm, the concentration of total phenols is deduced from a calibration curve established with gallic acid and the results were expressed in mg of gallic acid equivalent per g dried extract (mg GAE/ g extract).

### 2.5. Quantification of Flavonoids

The content of total flavonoids was determined according to the method described by Djeridane et al. [10]. Thereby, the extract was mixed (500 μL) with 500 μL of 2% aluminum chloride. The absorbance of the mixture is measured at 430 nm, after 10 min of incubation. The flavonoid concentrations were expressed in mg equivalent quercetin per g dried extract (mg QE/g extract) with reference to a calibration curve.

### 2.6. Antioxidant Activities

#### 2.6.1. Evaluation of Antioxidant Activity by β-Carotene Bleaching Test

The antioxidant activity of the extracts was evaluated using the *β*-carotene-linoleic acid system described by Miller [11] with a slight modification. Dissolve 0.5 mg of *β*-carotene in 1 mL of chloroform. The solution obtained was introduced into a flask containing a mixture of 25 µL of linoleic acid and 200 mg of Tween 40. After evaporation of the chloroform under vacuum, 100 mL of distilled water saturated with oxygen were added by vigorous stirring. From this new solution, 4 mL was transferred to different test tubes containing different concentrations of the sample in ethanol. As soon as the emulsion was added to each tube, the absorbance of the zero time was measured at 470 nm, using a spectrophotometer. The emulsion system was incubated for 2 h at 50 °C. A negative control, free of *β*-carotene, was prepared for background subtraction. The bleaching rate (R) of *β*-carotene was calculated according to the following equation: R = ln_a/b_/t. the natural log, a is the absorbance at zero time, b is the absorbance at time t (120 min). Antioxidant activity (AA) was calculated in terms of percent inhibition versus control, using the following equation:

*% inhibition* = [*R control* − *R sample/R control*] × 100.

Quercetin, BHT, and *α*-tocopherol have been used as antioxidant standards for the comparison.

#### 2.6.2. DPPH Free Radical Scavenging Test

The anti-radical activity against DPPH of the studied extracts was measured by the DPPH test described by Blois [12] with a slight modification. Briefly a 0.1 mM solution of DPPH in methanol was prepared and 4 mL of this prepared solution were added to 1 mL of sample solutions in methanol at different concentrations. After 30 min of incubation in the dark at room temperature, the absorbance is measured at 517 nm. Lower absorbance of the reaction mixture indicated greater free radical scavenging activity. The antioxidant activity was expressed as a percentage of DPPH radical inhibition, and calculated from the following equation:

*% inhibition* = [*A control* − *A sample/A control*] × 100.

The IC_50_ value (the inhibitory concentration of the extract necessary to decrease the initial concentration of the DPPH radical at 50%) was calculated from the percentage plot of the trapping effect of the different concentrations of each extract [13]. We deduced the anti-radical activity of the extracts by calculating the inverse of the IC_50_ values found [14], by the following formula: ARA = 1/IC_50_. Quercetin, BHT, and *α*-tocopherol have been used as antioxidant standards for the comparison of activity.

#### 2.6.3. ABTS Radical Cation Reduction Test

The anti-radical activity against the radical ABTS^+^ of the studied extracts was determined according to the method of Re et al. [15] with slight modification. In this test, the radical cation ABTS^+^ is generated by mixing 7 mM ABTS in H_2_O and 2.45 mM Potassium Persulfate. The mixture is then stored in the dark at room temperature for 12 h. The oxidation of ABTS^+^ started immediately, but the absorbance was not maximal and stable until more than 6 h had elapsed. The radical cation was stable in this form for more than 2 days with storage in the dark at room temperature. Before use, the ABTS^+^ solution was diluted with ethanol to obtain an absorbance of 0.700 ± 0.02 at 734 nm. Then 2 mL of ABTS^+^ solution was added to 1 mL of sample solution in ethanol at different concentrations (5–50 mg/mL). After 30 min, the percent inhibition at 734 nm was calculated for each concentration based on a blank absorbance (methanol). The ABTS^+^ scanning capability was calculated using the following equation:

*% inhibition* = [*Abs control* − *Abs sample/Abs control*] × 100

where the Abs controls are ABTS solution absorbance plus methanol, and the Abs sample is ABTS absorbance plus extract or standard. The IC_50_ value is calculated for each sample and compared with quercetin, BHT, and *α*-tocopherol, which were used as antioxidant standards for activity comparison.

#### 2.6.4. Cupric Reducing Antioxidant Capacity (CUPRAC) Test

The cupric reductive antioxidant capacity was determined according to the method of Apaket al. [16] with a slight modification. In each well, in a 96-well plate, 50 μL of 10 mM Cu (II) solution, 50 μL of 7.5 Mm neocuprone and 60 μL of NH_4_Ac buffer (1 M, pH 7.0) were added. 40 μL extracts at different concentrations were added to the initial mixture to obtain the final volume of 200 μL. After 1 h, the absorbance at 450 nm was recorded against a reagent blank using a 96-well microplate reader. The results were given as A_0.50_ (µg/mL), which corresponds to the concentration providing 0.500 absorbance. The concentration of the sample providing 0.50 absorbance (A_0.50_) was calculated from the graph of the absorbance of cupric reductive antioxidant capacity. BHT and *α*-tocopherol were used as antioxidant standards for comparing the activity.

#### 2.6.5. Ferrous Ions Chelating Test

The chelating activity of the Fe^2+^ extracts was measured using Ferrin [17] with slight modifications. The extract solution (80 μL dissolved in ethanol at different concentrations) was added to 40 μL of 0.2 mM FeCl_2_. The reaction was initiated by the addition of 80 μL of 0.5% ferene. The mixture was stirred vigorously and left at room temperature for 10 min. After the mixture reached equilibrium, the absorbance was measured at 593 nm. The chelating activity was calculated using the following equation:

*% of metal chelation activity* = [*A control* − *A sample/A control*] × 100

where A control is the absorbance of the sample-free control and A sample is the absorbance of the sample in the presence of the chelator. The concentration of extract providing 50% of metal chelation activity (IC_50_) was calculated from the graph of the percentage of Fe^2+^ chelation effects relative to the concentration of extract. EDTA and quercetin were used as antioxidant standards for the comparison of the activity.

### 2.7. Statistical Analysis

All data of antioxidant activities tests were the average of three analyses. The data were recorded as mean ± standard deviation. Significant differences between means were determined by student’s-t test and *p* values <0.05 were considered as significant results.

## 3. Results and Discussion

### 3.1. Analysis of LC–MS/MS

According to the results of LC-MS/MS analysis, the analyzed extracts were rich in phenolic acids and flavonoids. A total of 12 phenolic acids (Gallic acid, protocatechic acid, chlorogenic acid, vanillic acid, caffeic acid, syringic acid, salicylic acid, ferulic acid, sinapicacid, rosmarinic acid, 4-OH-benzoic acid and *p*-coumaric acid), seven flavonoids (rutin, hesperidin, isoquercitrin, rhoifolin, quercitrin, apigetrin and apigenin), two non-phenolic organic acids (Quinic acid, malic acid), one phenolic aldehyde (vanillin), and one benzopyrone (coumarin)were identified in the analyzed plant samples(Table 3).

**Figure 3:**
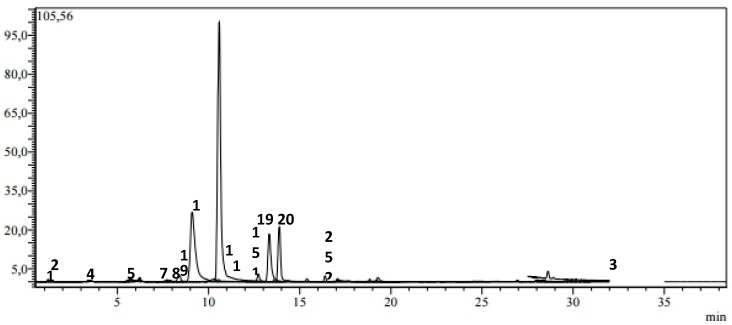
LC-MS/MS chromatogram of EA extract of *H. laevigata* var. *hipponensis*.

**Figure 4:**
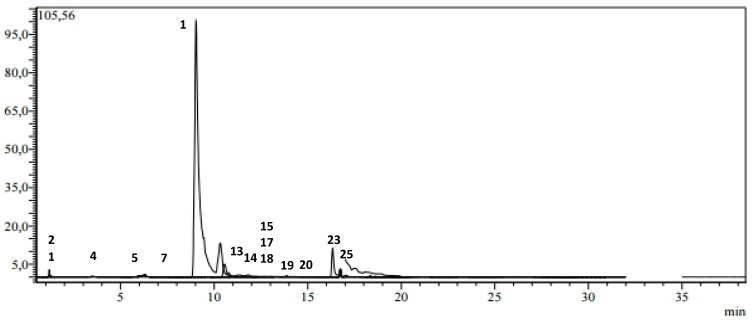
LC-MS/MS chromatogram of BuOH extract of *H. laevigata* var. *hipponensis*.

**Figure 5:**
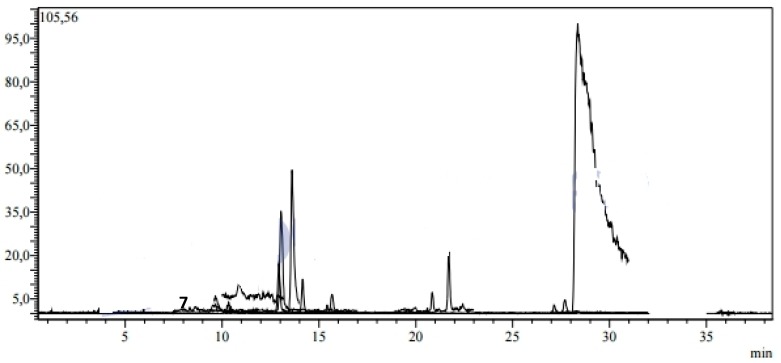
LC-MS/MS chromatogram of DCM extract of *H. laevigata* var. *hipponensis*.

The ethylacetate fraction (EA) showed the highest values with the presence of 20 phenolic compounds (Figure 3), followed by n-butanol (BuOH) and dichloromethane(DCM) fractions with 15 and nine phenolic compounds, respectively (Figure 4 and Figure 5).

The LC-MS analysis of the ethyl acetate extract (EA)revealed the presence of quinic acid, chlorogenic acid, caffeic acid, ferulicacid, *p*-coumaric acid, syringic acid, and 4-OH-benzoic acid which showed the highest concentrations (9633.02, 2689.03, 1537.29, 1319.88, 1235.76, 1263.17, and 912.26 μg/g of extract, respectively). Meanwhile, rhoifolin, apigenin, rosmarinic acid, apigetrin, salicylic acid, hesperidin, quercitrin and isoquercitrin (28.58, 35.5, 39.58, 54.62, 69.03, 77.24, 83.47, 98.55 μg/g of extract, respectively) were found with the lowest values.

The phenolic compounds were reported to have a beneficial effect on health and can also be exploited for phyto-pharmaceutical applications because of their biological properties [18]. Among the main compounds found in our extracts, gallic acid is one of the most important phenolic compounds, due to its antineoplastic, bacteriostatic, anti-melanogenic, antioxidant, and anticancer properties [19]. However, chlorogenic, caffeic, and ferulic acids proved to have antioxidant and antimicrobial activities [18]. 

Whereas flavonoids can treat different diseases, such as viral, inflammatory, liver, allergic, thrombotic, and cancer [18,19,20,21], the flavonoids which we found in the plant were reported to have various biological activities. For example, rutin exhibited beneficial effects such as antioxidants, anti-allergic, antiviral, anti-inflammatory, anti-atherosclerosis by inhibiting platelet aggregation as well as anticancer activity. It has also been suggested to play a protective role in cardiovascular diseases and liver [20,21]. Meanwhile, apigetrin is a substance applied in the treatment of diabetes and cancer [22,23].

### 3.2. Total Phenolic and Flavonoid Contents

The results of the total phenolic contents (Table 4) of the three extracts of *H. laevigata* var. *hipponensis* showed that the ethyl acetate (EA) and n-butanol (BuOH) extracts have the highest value with 202.86 ± 14.64 and 200 ± 10.93 GAE/g extract, respectively. Also, the total flavonoid content (Table 4) of the BuOH extract (46.76 ± 0.36 QE/g of extract) was greater than that of EA and DCM extracts (17.92 ± 0.12 and 16.28 ± 0.16 QE/g extract, respectively).

### 3.3. Antioxidant Activities

In the present work, the antioxidant activity was determined by five methods (Table 5). For β-carotene test, good activity was found in the three extracts (IC_50_ value of 5.02 ± 0.95, 5.66 ± 2.03 and 7.60 ± 4.37 for dichloromethane, *n*-butanol and ethyl acetate, respectively), it was better than that of catechin (8.79 ± 0.89 µg/mL) and higher of α-tocopherol, BHT and quercetin (2.10 ± 0.08, 1.34 ± 0.04 and 1.81 ± 0.11 µg/mL).

For the DPPH test, maximum scavenging activity was found in *n*-butanol extract (IC_50_ value: 8.12 ± 1.47 µg/mL) followed by ethyl acetate extract (IC_50_ value: 8.70 ± 1.87 µg/mL). Dichloromethane extract showed a bit important activity (47.24 ± 0.11 µg/mL). Studies reported that anti-radical activity is correlated with the level of polyphenols and flavonoids in medicinal plant extract [24,25,26,27,28,29].

In the ABTS+ method, the ethyl acetate extract(EA) exhibited the highest activity with an IC_50_ value of 4.32 ± 0.09 µg/mL among all extract (IC_50_ value of DCM extract 13.10 ± 0.97 and BuOH extract 15.02 ± 0.73 µg/mL) in comparison to α-tocopherol and BHT (4.31 ± 0.10 and 4.10 ± 0.06 µg/mL). The results proved that the extracts have the ability to trap the various free radicals in the different systems, indicating that they can be useful for therapeutic agents and for the treatment of radical-related pathological lesions [30].

Results of the CUPRAC test of EA and BuOH extracts exhibited a higher activity (A_0.50_ value: 1.48 ± 0.33 and 3.00 ± 0.98 µg/mL, respectively) than those of standards (BHT with 3.80 ± 0.00 and α-tocopherol with 10.20 ± 0.01 µg/mL).The results we found are similar to those of Gorinsteinet al. [31], who say that the highest capacities of polyphenolic compounds are measured with CUPRAC, and they are also similar to the results of Prior et al. [32], who found that the CUPRAC method showed the highest antioxidant activities compared with other antioxidants tests. 

For the ferrous ions chelation test, all extracts were not active.

## 4. Conclusions

This study was performed to investigate the chemical composition of phenolic compounds in dichloromethane, ethyl acetate, and *n*-butanol extracts of *H. laevigata* var. *hipponensis* by the liquid chromatography-mass spectrometry (LC-MS/MS) technique. The LC-MS/MS lead a total of 23 chemical compounds in the three extracts (EA with 20 compounds, BuOH with 15 compounds, and DCM with nine compounds). Chlorogenic acid, caffeic acid, ferulic acid, *p*-coumaric acid, syringic acid, and 4-OH-benzoic were the major phenolic compounds detected. The total phenolic contents indicated that EA fraction and BuOH presented the highest value. The flavonoid content showed that BuOH exhibited the highest value. The antioxidant activities of the tested extracts showed a positive result with the *β*-carotene bleaching method, DPPH radical scavenging activity, ABTS cation radical scavenging activity, and cupric reducing antioxidant capacity. However, the ferrous iron chelation assay showed a negative result. When we compared the antioxidant activities of the three extracts, we found that the ethyl acetate extract was more potent than the n-butanol and dichloromethane extracts. Finally, we recommend the continuation our work in the future to evaluate more biological activities in vitro and in vivo, and to isolate the chemical compounds of the plant.

## Figures and Tables

**Table 1:** HPLC–MS/MS acquisition parameters used for the analysis of the 37 marker compounds in the extracts of *Hypochaerislaevigata* var. *hipponensis*.

No.	Compounds	Retention Time (min)	Scan Type	Polarity or (ESI Mode)	Precursor Ion [M-H]^−^ (*m/z*)	MS^2^Fragments or Product Ions (*m/z*)
1	Quinic acid	1.13	MRM	Negative	190.95	85.3–93.3
2	Malic acid	1.23	MRM	Negative	133.00	115.2–71.3
3	Fumaric acid	1.48	MRM	Negative	115.00	71.4
4	Gallic acid	3.00	MRM	Negative	168.85	125.2–79.2
5	Protocatechic acid	4.93	MRM	Negative	152.95	108.3
6	Pyrocatechol	6.48	MRM	Negative	109.00	108.35–91.3
7	Chlorogenic acid	7.13	MRM	Negative	353.15	191.2
8	4-OH-Benzoic acid	7.39	MRM	Negative	136.95	93.3–65.3
9	Vanillic acid	8.57	MRM	Negative	166.90	152.3–108.3
10	Caffeic acid	8.80	MRM	Negative	178.95	135.2–134.3
11	Syringic acid	9.02	MRM	Negative	196.95	182.2–167.3
12	Vanillin	10.87	MRM	Negative	151.00	1363–92.2
13	Salicylic acid	11.16	MRM	Negative	136.95	93.3–65.3
14	*p*-Coumaric acid	11.53	MRM	Negative	162.95	119.3–93.3
15	Rutin	12.61	MRM	Negative	609.05	300.1–271.1
16	Ferulic acid	12.62	MRM	Negative	192.95	178.3
17	Sinapic acid	12.66	MRM	Negative	222.95	208.3–149.2
18	Hesperidin	12.67	MRM	Negative	609	301.1
19	Isoquercitrin	13.42	MRM	Negative	463.00	300.1–271.1
20	Rosmarinic acid	14.54	MRM	Negative	359.00	161.2–197.2
21	Nicotiflorin	14.68	MRM	Negative	593.05	285.1–255.2
22	*α*-Coumaric acid	15.45	MRM	Negative	162.95	119.4–93.3
23	Rhoifolin	16.11	MRM	Negative	577.05	269.2–211.1
24	Quercitrin	16.41	MRM	Negative	447.15	301.1–255.1
25	Apigetrin	16.59	MRM	Negative	431.00	268.2–239.2
26	Coumarin	17.40	MRM	Negative	147.05	91.0–103.2
27	Myricetin	18.72	MRM	Negative	317.00	179.2–151.3
28	Fisetin	19.30	MRM	Negative	284.95	135.2–121.3
29	Cinnamic acid	25.61	MRM	Negative	147.00	103.15–77.3
30	Liquiritigenin	25.62	MRM	Negative	254.95	119.3–135.1
31	Quercetin	28.17	MRM	Negative	300.90	151.2–179.2
32	Luteolin	28.27	MRM	Negative	284.75	133.2–151.2
33	Naringenin	30.68	MRM	Negative	270.95	151.2–119.3
34	Apigenin	31.43	MRM	Negative	268.95	117.3–151.2
35	Hesperetin	31.76	MRM	Negative	300.95	164.2–136.2
36	Kaempferol	31.88	MRM	Negative	284.75	255.1–117.3
37	Chrysin	36.65	MRM	Negative	252.95	143.3–119.4

**Table 2:** Concentration range, linearity (R^2^), Limits of Detection (LODs), Limits of Quantification (LOQs) and percentages of recoveries of the analysed 37 compounds by LC–MS/MS.

N	Compounds	Conc. Range (Linearity Range) (μg/mL)	R^2^	LOD (μg/mL)	LOQ (μg/mL)	Inter-Day (*n* = 3) RSD (%)	Intra-Day (*n* = 3) RSD (%)	Recovery % (*n* = 3)		U (%)
								Inter-Day	Intra-Day	
1	Quinic acid	0.250–10	0.996	0.075	0.079	0.259	0.274	100.28	98.77	0.0082
2	Malic acid	0.250–10	0.999	0.055	0.067	0.477	0.527	101.26	99.83	0.0113
3	Fumaric acid	0.10–5	0.997	0.028	0.034	0.536	0.460	99.74	99.86	0.0124
4	Gallic acid	0.250–10	0.998	0.095	0.106	1.601	01.443	100.00	100.45	0.0282
5	Protocatechic acid	0.100–5	0.995	0.028	0.031	1.236	1.296	99.40	101.07	0.0411
6	Pyrocatechol	1–20	0.996	0.261	0.278	1.313	1.339	99.98	99.93	0.0235
7	Chlorogenic acid	0.025–1	0.998	0.006	0.008	0.058	0.076	100.80	99.96	0.0069
8	4-OH-Benzoic acid	0.250–10	0.998	0.033	0.038	1.284	1.538	99.66	100.05	0.0289
9	Vanillic acid	0.1–20	0.999	0.122	0.139	0.528	0.619	100.09	104.09	0.0508
10	Caffeic acid	0.025–1	0.998	0.018	0.022	1.454	1.469	100.91	98.82	0.0354
11	Syringic acid	0.1–20	0.996	0.021	0.233	1.049	1.345	99.92	99.97	0.0238
12	Vanillin	0.250–10	0.998	0.044	0.053	0.696	0.793	99.67	99.61	0.0280
13	Salicylic acid	0.025–1	0.989	0.005	0.006	1.016	1.242	100.98	99.01	0.0329
14	*p*-Coumaric acid	0.025–1	0.992	0.007	0.009	1.820	1.727	100.61	101.22	0.0516
15	Rutin	0.025–1	0.997	0.005	0.006	0.473	0.624	100.99	98.01	0.0159
16	Ferulic acid	0.250–10	0.997	0.036	0.042	0.708	0.619	99.98	100.28	0.0494
17	Sinapic acid	0.250–10	0.992	0.078	0.086	1.446	1.517	100.16	99.96	0.0281
18	Hesperidin	0.025–1	0.998	0.003	0.004	0.945	1.126	101.73	101.26	0.0262
19	Isoquercitrin	0.025–1	0.999	0.005	0.006	0.682	0.515	100.59	100.72	0.0133
20	Rosmarinic acid	0.100–5	0.994	0.006	0.008	2.014	1.751	99.20	103.43	0.0713
21	Nicotiflorin	0.100–5	0.991	0.022	0.025	0.737	0.875	102.55	100.97	0.0276
22	*α*-Coumaric acid	0.025–1	0.999	0.024	0.031	2.730	2.566	98.34	99.06	0.0513
23	Rhoifolin	0.100–5	0.999	0.023	0.027	0.747	1.528	101.04	101.73	0.0941
24	Quercitrin	0.100–5	0.999	0.022	0.025	1.528	2.320	99.72	100.62	2.0079
25	Apigetrin	0.025–1	0.993	0.005	0.006	1.797	1.607	101.39	100.41	0.0597
26	Coumarin	1–20	0.994	0.208	0.228	1.306	1.239	99.94	100.08	0.0237
27	Myricetin	0.250–10	0.999	0.053	0.057	0.652	0.711	99.98	100.04	0.0126
28	Fisetin	0.250–10	0.991	0.054	0.051	0.557	0.820	99.87	100.03	0.0148
29	Cinnamic acid	5–20	0.996	0.821	0.859	0.648	0.816	100.05	99.92	0.0143
30	Liquiritigenin	0.025–1	0.996	0.005	0.006	1.849	1.738	100.33	99.95	0.0341
31	Quercetin	0.100–5	0.990	0.023	0.028	1.589	1.360	98.47	100.10	0.0543
32	Luteolin	0.025–1	0.997	0.005	0.006	0.575	0.696	100.77	99.52	0.0174
33	Naringenin	0.025–1	0.995	0.005	0.006	2.054	2.019	99.88	101.00	0.0521
34	Apigenin	0.025–1	0.990	0.005	0.006	2.304	2.204	101.44	101.33	0.0650
35	Hesperetin	0.025–1	0.997	0.005	0.006	3.209	2.605	98.85	99.43	0.0562
36	Kaempferol	1–20	0.992	0.206	0.214	1.436	1.070	99.97	99.85	0.0209
37	Chrysin	0.02–1	0.993	0.005	0.006	0.490	0.630	100.33	100.43	2.0083

RSD %: relative standard deviation. U (%): uncertainty Percent at 95% confidence level (k = 2).

**Table 3:** Quantitative determination of 37 phenolic compounds in the extracts of *Hypochaeris laevigata* var. *hipponensis* (μg/g extract) by LC-MS/MS.

N	Compounds	DCM	EA	BuOH
1	Quinic acid	N.I	9633.02	21,606.73
2	Malic acid	N.I	349.27	750.10
3	Fumaric acid	N.I	N.I	N.I
4	Gallic acid	N.I	223.26	115.09
5	Protocatechic acid	N.I	547.25	54.77
6	Pyrocatechol	N.I	N.I	N.I
7	Chlorogenic acid	9.39	2689.03	11,956.23
8	4-OH-Benzoic acid	N.I	912.26	N.I
9	Vanillic acid	148.3	1027.7	N.I
10	Caffeic acid	1.82	1537.29	98.47
11	Syringic acid	N.I	1235.76	N.I
12	Vanillin	58.32	N.I	N.I
13	Salicylic acid	17.5	69.03	3.54
14	*p*-Coumaric acid	10.38	1263.17	31.72
15	Rutin	N.I	198.71	1348.25
16	Ferulicacid	224.36	1319.88	N.I
17	Sinapic acid	N.I	N.I	948.68
18	Hesperidin	N.I	77.24	74.7
19	Isoquercitrin	N.I	98.55	76.3
20	Rosmarinic acid	N.I	39.58	12.49
21	Nicotiflorin	N.I	N.I	N.I
22	*α*-Coumaric acid	N.I	N.I	N.I
23	Rhoifolin	N.I	28.58	250.08
24	Quercitrin	N.I	83.47	N.I
25	Apigetrin	N.I	54.62	18.07
26	Coumarin	1171.49	N.I	N.I
27	Myricetin	N.I	N.I	N.I
28	Fisetin	N.I	N.I	N.I
29	Cinnamic acid	N.I	N.I	N.I
30	Liquiritigenin	N.I	N.I	N.I
31	Quercetin	N.I	N.I	N.I
32	Luteolin	N.I	N.I	N.I
33	Naringenin	N.I	N.I	N.I
34	Apigenin	151.43	35.5	N.I
35	Hesperetin	N.I	N.I	N.I
36	Kaempferol	N.I	N.I	N.I
37	Chrysin	N.I	N.I	N.I

N.I: Not Identified.

**Table 4:** Total phenolic and flavonoid contents of the extracts of *H. laevigata* var. *hipponensis*.

Extracts	Total Phenols ^a^	Flavonoids ^b^
**DCM**	184.07 ± 0.17	16.28 ± 0.16
**EA**	202.86 ± 14.64	17.92 ± 0.12
**BuOH**	200 ± 10.93	46.76 ± 0.36

^a^: mg Gallic Acid Equivalent/g extract; ^b^: mg Quercetin Equivalent/g extract.

**Table 5:** Antioxidant activities of the three extracts of *H. laevigata* var. *hipponensis*.

Extract	β-Carotene IC50 (µg/mL)	DPPH IC50 (µg/mL)	ABTS+ IC50 (µg/mL)	CUPRAC A0.50 (µg/mL)	Fe^+2^ Chelation IC_50_ (µg/mL)
**Dichloromethane**	5.02 ± 0.95	47.24 ± 0.11	13.10 ± 0.97	16.86 ± 3.02	>800
**Ethyl acetate**	7.60 ± 4.37	8.70 ± 1.87	4.32 ± 0.09	1.48 ± 0.33	>800
***n*-Butanol**	5.66 ± 2.03	8.12 ± 1.47	15.02 ± 0.73	3.00 ± 0.98	>800
**(+)-Catechin ^a^**	8.79 ± 0.89	4.32 ± 0.15	1.16 ± 0.02	NT	NT
**Quercetin ^a^**	1.81 ± 0.11	2.07 ± 0.10	1.18 ± 0.03	NT	NT
**α-Tocopherol ^a^**	2.10 ± 0.08	7.31 ± 0.17	4.31 ± 0.10	10.20 ± 0.01	NT
**BHT ^a^**	1.34 ± 0.04	45.4 ± 0.47	4.10 ± 0.06	3.80 ± 0.00	NT
**EDTA ^a^**	NT	NT	NT	NT	6.50 ± 0.07
**Ascorbic acid ^a^**	NT	NT	NT	NT	NT

**^a^** Standards compounds; NT: Not Tested.

## References

[1] Hamel T. (2013). Contribution à l’étude de l’endémisme chez les végétaux vasculaires dans la péninsule de l’Edough (Nord-Est algérien). Ph.D. Thesis.

[2] Stebbins G.L., Arnold E. (1971). Chromosomal changes, genetic recombination and speciation. Chromosomal Evolution in Higher Plants.

[3] Quézel P., Santa S. (1962). Nouvelle Flore d’Algérie et des Régions Désertiques Méridionales.

[4] Jamuna S., Paulsamy S., Karthika K. (2014). Phytochemical analysis and evaluation of leaf and root parts of the medicinal herb, *Hypochaeris radicata* L. for in vitro antioxidant activities. Asian Pac. J. Tro. Bio..

[5] Jamuna S., Paulsamy S., Karthika K. (2012). Screening of in vitro antioxidant activity of methanolic leaf and root extracts of *Hypochaeris radicata* L. (Asteraceae). J. Appl. Pharm. Sci..

[6] Jamuna S., Paulsamy S., Karthika K. (2013). In vitro antibacterial activity of leaf and root extracts of *Hypochaeris radicata* L. (Asteraceae): A medicinal plants pecies in habiting the high hills of Nilgiris, the Western Ghats. Int. J. Pharm. Pharm. Sci..

[7] Jamuna S., Paulsamy S., Karthika K. (2013). In vitro antifungal activity of leaf and root extracts of the medicinal plant, *Hypochaeris radicata* L.. Int. J. Pharm. Pharm. Sci..

[8] Pullaiah T. (2006). Encyclopedia of World Medicinal Plants.

[9] Li R., Guo M., Zhang G., Xu X., Li K. (2007). Neuroprotection of Nicotiflorin in Permanent Focal CerebralIschemia and in Neuronal Cultures. Biol. Pharm. Bull..

[10] Djeridane A., Yousfi M., Nadjemi B., Boutassouna D., Stocker P., Vidal N. (2006). Antioxidant activity of some Algerian medicinal plants extracts containing phenolic compounds. Food. Chem..

[11] Miller E.D. (1971). Isolation and characterization of the cyanogenbromide peptides from the alpha. 1 (II) chain of chick cartilage collagen. Biochemistry.

[12] Blois M.S. (1958). Antioxidant Determinations by the Use of a Stable Free Radical. Int. J. Sci. Nat..

[13] Scherer R., Godoy H.T. (2009). Antioxidant activity index (AAI) by the 2,2-diphenyl-1-picrylhydrazyl method. Food Chem..

[14] Maisuthisakul P., Suttajit M., Pongsawatmanit R. (2007). Assessment of phenolic content and free radical-scavenging capacity of some Thai indigenous plants. Food Chem..

[15] Re R., Pellegrini N., Proteggente A., Pannala A., Yang M., Rice-Evans C. (1999). Antioxidant activity applying an improved ABTS radical cation decolorization assay. Free Radic. Bio. Med..

[16] Apak R., Güçlü K., Özyürek M., Karademir S.E. (2004). Novel total antioxidant capacity index for dietary polyphenols and vitamins C and E, using their cupric ion reducing capability in the presence of neocuproine: CUPRAC Method. J. Agric. Food Chem..

[17] Decker E.A., Welch B. (1990). Roleof ferritin as a lipid oxidation catalystin muscle food. J. Agric. Food Chem..

[18] Heleno S.A., Martins A., Queiroz M.J.R., Ferreira I.C. (2015). Bioactivity of phenolic acids: Metabolites versusparent compounds: A review. Food Chem..

[19] Hur J.Y., Soh Y., Kim B.-H., Suk K., Sohn N.W., Kim H.C., Kwon H.C., Lee K.R., Kim S.Y. (2001). Neuroprotective and Neurotrophic Effects of Quinic Acids from Asterscaberin PC12Cells. Biol. Pharm. Bull..

[20] Tapas A.R., Sakarkar D.M., Kakde R.B. (2008). Flavonoids as nutraceuticals: A review. Trop. J. Pharm. Res..

[21] Tripoli E., LaGuardia M., Giammanco S., DiMajo D., Giammanco M. (2007). *Citrus* flavonoids: Molecular structure, biological activity and nutritional properties: A review. Food Chem..

[22] Tsolmon S., Nakazaki E., Han J., Isoda H. (2011). Apigetrin induces erythroid differentiation of human leukemia cells K562: Proteomics approach. Mol. Nutr. Food Res..

[23] Rao Y.K., Lee M.J., Chen K., Lee Y.-C., Wu W.-S., Tzeng Y.-M. (2011). Insulin-Mimetic Action of Rhoifolin and Cosmosiin Isolated from *Citrus grandis* (L.) Osbeck Leaves: Enhanced Adiponectin Secretion and Insulin Receptor Phosphorylation in 3T3-L1 Cells. Evid. Based Complementary Altern. Med..

[24] Mariod A.A., Ibrahim R.M., Ismail M., Ismail N. (2009). Antioxidant activity and phenolic content of phenolic rich fractions obtained from black cumin (*Nigella sativa*) seed cake. Food Chem..

[25] Martinez J., Nieto G., Castillo J., Ros G. (2014). Influence of in vitro gastrointestinal digestion and/ orgrape seed extract addition on antioxidant capacity of meatemul-sions. Lebensmittel- Wissenschaftund-Technologie LWT.

[26] Nieto G., Bañon S., Garrido M.D. (2012). Incorporation of thyme leaves in the diet of pregnant and lactating ewes: Effect on the fatty acid profile of lamb. Small Rum. Res..

[27] Nieto G., Bañon S., Garrido M.D. (2012). Administration of distillate thyme leaves in to the diet of Segureña ewes: Effect on lambme at quality. Animal.

[28] Nieto G. (2013). Incorporation of by- products of rosemary and thyme in the diet of ewes: Effect on the fatty acid profile of lamb. Eur. Food Res. Technol..

[29] Martínez L., Jongberg S., Ros G., Skibsted L.H., Nieto G. (2020). Plant derived ingredients rich in nitrates or phenolics for protection of pork against protein oxidation. Food Res. Int..

[30] Wang M., Li J., Rangarajan M., Shao Y., LaVoie E.J., Huang T.-C., Ho C.-T. (1998). Antioxidative phenolic compounds from sage (*Salvia officinalis*). J. Agric. Food Chem..

[31] Gorinstein S., Leontowicz M., Leontowicz H., Najman K., Namiesnik J., Park Y.-S., Jung S.-T., Kang S.-G., Trakhtenberg S. (2006). Supplementation of garlic lowers lipids and increases antioxidant capacity in plasma of rats. Nutr. Res..

[32] Prior R.L., Wu X., Schaich K. (2005). Standardized methods for the determination of antioxidant capacity and phenolics in foods and dietary supplements. J. Agric. Food Chem..

